# Assessment of Disparities in Diabetes Mortality in Adults in US Rural vs Nonrural Counties, 1999-2018

**DOI:** 10.1001/jamanetworkopen.2022.32318

**Published:** 2022-09-20

**Authors:** Sagar B. Dugani, Christina M. Wood-Wentz, Michelle M. Mielke, Kent R. Bailey, Adrian Vella

**Affiliations:** 1 Division of Hospital Internal Medicine, Mayo Clinic, Rochester, Minnesota; 2 Division of Health Care Delivery Research, Kern Center for the Science of Health Care Delivery, Mayo Clinic, Rochester, Minnesota; 3 Knowledge and Evaluation Research Unit, Mayo Clinic, Rochester, Minnesota; 4 Department of Quantitative Health Sciences, Mayo Clinic, Rochester, Minnesota; 5 Now with Department of Epidemiology and Prevention, Wake Forest University School of Medicine, Winston-Salem, North Carolina; 6 Division of Endocrinology, Mayo Clinic, Rochester, Minnesota

## Abstract

**Importance:**

US rural vs nonrural populations have striking disparities in diabetes care. Whether rurality contributes to disparities in diabetes mortality is unknown.

**Objective:**

To examine rates and trends in diabetes mortality based on county urbanization.

**Design, Setting, and Participants:**

In this observational, cross-sectional study, the US Centers for Disease Control and Prevention Wide-Ranging Online Data for Epidemiologic Research database was searched from January 1, 1999, to December 31, 2018, for diabetes as a multiple cause and the underlying cause of death among residents aged 25 years or older in US counties. County urbanization was categorized as metro, medium-small, and rural. Weighted multiple linear regression models and jackknife resampling, with a 3-segment time component, were used. The models included exposures with up to 3-way interactions and were age standardized to the 2009-2010 population. The analyses were conducted from July 1, 2020, to February 1, 2022.

**Exposures:**

County urbanization (metro, medium-small, or rural), gender (men or women), age group (25-54, 55-74, or ≥75 years), and region (Midwest, Northeast, South, or West).

**Main Outcomes and Measures:**

Annual diabetes mortality rate per 100 000 people.

**Results:**

From 1999-2018, based on 4 022 238 309 person-years, diabetes was a multiple cause of death for 4 735 849 adults aged 25 years or older. As a multiple cause, diabetes mortality rates in 2017-2018 vs 1999-2000 were highest and unchanged in rural counties (157.2 [95% CI, 150.7-163.7] vs 154.1 [95% CI, 148.2-160.1]; *P* = .49) but lower in medium-small counties (123.6 [95% CI, 119.6-127.6] vs 133.6 [95% CI, 128.4-138.8]; *P* = .003) and urban counties (92.9 [95% CI, 90.5-95.3] vs 109.7 [95% CI, 105.2-114.1]; *P* < .001). In 2017-2018 vs 1999-2000, mortality rates were higher in rural men (+18.2; 95% CI, 14.3-22.1) but lower in rural women (−14.0; 95% CI, −17.7 to −10.3) (*P* < .001 for both). In the 25- to 54-year age group, mortality rates in 2017-2018 vs 1999-2000 showed a greater increase in rural counties (+9.4; 95% CI, 8.6-10.2) compared with medium-small counties (+4.5; 95% CI, 4.0-5.0) and metro counties (+0.9; 95% CI, 0.4-1.4) (*P* < .001 for all). Of all regions and urbanization levels, the mortality rate in 2017-2018 vs 1999-2000 was higher only in the rural South (+13.8; 95% CI, 7.6-20.0; *P* < .001).

**Conclusions and Relevance:**

In this cross-sectional study, US rural counties had the highest overall diabetes mortality rate. The determinants of persistent rural disparities, in particular for rural men and for adults in the rural South, require investigation.

## Key Points

**Question:**

In the US, do disparities exist in diabetes mortality based on county urbanization?

**Findings:**

In this cross-sectional study, in 2017-2018 vs 1999-2000, the age-standardized diabetes mortality rates per 100 000 people were unchanged in rural counties (157.2 vs 154.1) but significantly lower in medium-small counties (123.6 vs 133.6) and metro counties (92.9 vs 109.7). In 2017-2018 vs 1999-2000, the mortality rate was significantly higher in rural men (+18.2) but lower in rural women (−14.0).

**Meaning:**

These findings indicate that overall, US rural counties have persistently high diabetes mortality rates, with additional disparities based on gender.

## Teaser

This cross-sectional study of US residents examines subnational trends in diabetes mortality based on county urbanization.

## Introduction

Diabetes is a chronic condition that requires lifelong, longitudinal care. In the US, the prevalence of diabetes and access to diabetes care are affected by several factors, including rurality.^1,2,3^ The prevalence of diabetes is, in part, determined by trends in mortality. However, in rural populations, information is sparse on trends in diabetes mortality,^1^ thereby limiting the development of rural interventions to reduce mortality.

From 1980 to 2017, the age-standardized prevalence of diabetes was steady from 1980 to 1990, increased from 1990 to 2009, and then plateaued through 2017.^4^ During this period, the age-standardized incidence rate of diabetes increased from 1990 to 2007 and decreased through 2017.^4^ In comparison, the longitudinal trend in diabetes-related mortality rates is incompletely described. Furthermore, national trends in mortality rates, as reported for prevalence and incidence, do not necessarily reflect subnational trends, which may be more meaningful for targeted efforts to monitor, manage, and improve diabetes care.

To address these knowledge gaps, we used the US Centers for Disease Control and Prevention Wide-Ranging Online Data for Epidemiologic Research (CDC WONDER) data for January 1, 1999, to December 31, 2018, to examine rates and trends in diabetes mortality among adults based on county urbanization, gender, age group, and region. The objective of the study was to examine subnational trends in diabetes mortality to guide the development of rural interventions to reduce mortality.

## Methods

### Data Source

In this cross-sectional study, we extracted CDC WONDER data for 1999 to 2018 for diabetes as a top 20 contributor to death (multiple cause of death) and leading cause of death (underlying cause of death).^5^ We restricted the analysis to the 1999 to 2018 period because there are no county urbanization data before 1999 and data for 2018 were the last available at study initiation.^5^ The use of public data was deemed not human subjects research by the Mayo Clinic Institutional Review Board, and no patient informed consent was required. The study followed the Strengthening the Reporting of Observational Studies in Epidemiology (STROBE) reporting guideline.

We grouped mortality by region (Midwest, Northeast, South, or West), county urbanization (metro, medium-small, or rural), age group (25-54, 55-74, or ≥75 years), and gender (men or women) in 2-year groups (eg, 1999-2000).^5^ We did not stratify by race or ethnicity to avoid suppressed data and erroneous estimates. The CDC WONDER database suppresses numerator counts with fewer than 10 deaths; however, our grouping criteria did not generate suppressed data.

Mortality attributed to diabetes (type 1 and type 2) was based on *International Statistical Classification of Diseases and Related Health Problems, Tenth Revision* (*ICD-10*) codes E10 to E14. Type 2 diabetes accounts for 90% to 95% of diabetes cases, and our findings likely pertain to type 2 diabetes.^6,7^ We excluded individuals younger than 25 years, who have a higher prevalence of type 1 compared with type 2 diabetes.

### County Urbanization

We used a condensed version of the 2013 National Center for Health Statistics criteria: metro (large central metropolitan and large fringe metropolitan), medium-small (medium metropolitan and small metropolitan), and rural (micropolitan and noncore).^8,9^

### Statistical Analysis

We assessed models with different numbers of time segments (2, 3, and 4) and selected a 3-segment linear model with 2 change points based on significance of fit improvement (eMethods 1 in the Supplement). To determine the number and location of change points, we fit a main effects model (gender, age group, region, county urbanization, and time), varying the change points from 1999-2000 to 2017-2018. For diabetes as a multiple cause of death, the fits with the lowest mean square error were for change points at (1) 2003-2004 and 2011-2012 and (2) 2003-2004 and 2009-2010. Given the minimal difference in mean square values, we selected 2003-2004 and 2009-2010 as change points because model predictions were age standardized to the 2009-2010 population. The same change points were used for diabetes as the underlying cause of death. With this basic time structure, we fit models with up to 3-way interactions among the exposures and time variables (eMethods 1 in the Supplement). Assuming Poisson-like variation of death counts in each cell, weighted multiple linear regression was used to model the outcome (log [mortality rate]) that was reported as annual diabetes mortality rate (ADMR) per 100 000 people (eMethods 1 in the Supplement).

We modeled deaths by gender, age group, region, county urbanization, and year to evaluate the joint effects of, and interactions between, the exposures on mortality rates. To summarize temporal trends on the original scale of mortality rates, we derived unadjusted and adjusted estimates of mortality rates for the beginning and ending years, along with the 2 change points, and age standardized to the 2009-2010 population. Unadjusted estimates represent the hypothetical effect of the tabled variable, while allowing nontabled variables to vary as they did in the original data set. Adjusted estimates represent the hypothetical effects of the same tabled variables if the nontabled variables were equally distributed within each level of the tabled variables (eMethods 2 in the Supplement).

To evaluate whether variation in mortality time trends with respect to other exposures (ie, gender, age group, and region) accounted for the variation in mortality time trends across urbanization levels, we compared the F statistics for time × urbanization within models with and without inclusion of other time × exposures (eMethods 3 in the Supplement).

The SEs and 95% CIs for ADMR and comparisons between time points and urbanization levels were based on a jackknife procedure by individually omitting each of the 720 cells from model estimation, followed by aggregation of estimated deaths and age standardized to the 2009-2010 population. Statistical analysis was conducted using SAS software, version 9.4 (SAS Institute Inc) with statistical significance at a 2-tailed *P* < .05.

## Results

The analysis was based on 4 022 238 309 person-years in adults 25 years or older and 4 735 849 deaths with diabetes as a multiple cause of death (annual mean [SE] of 117.7 [0.05] deaths per 100 000 people). From 1999 to 2018, the population characteristics varied by county urbanization (eTable 1 in the Supplement). With increasing rurality, there was a lower proportion of women (52.1% in metro, 51.9% in medium-small, and 51.0% in rural counties) and a higher proportion of adults 55 years or older (34.9% in metro, 39.0% in medium-small, and 42.9% in rural counties).

**Figure 1.**
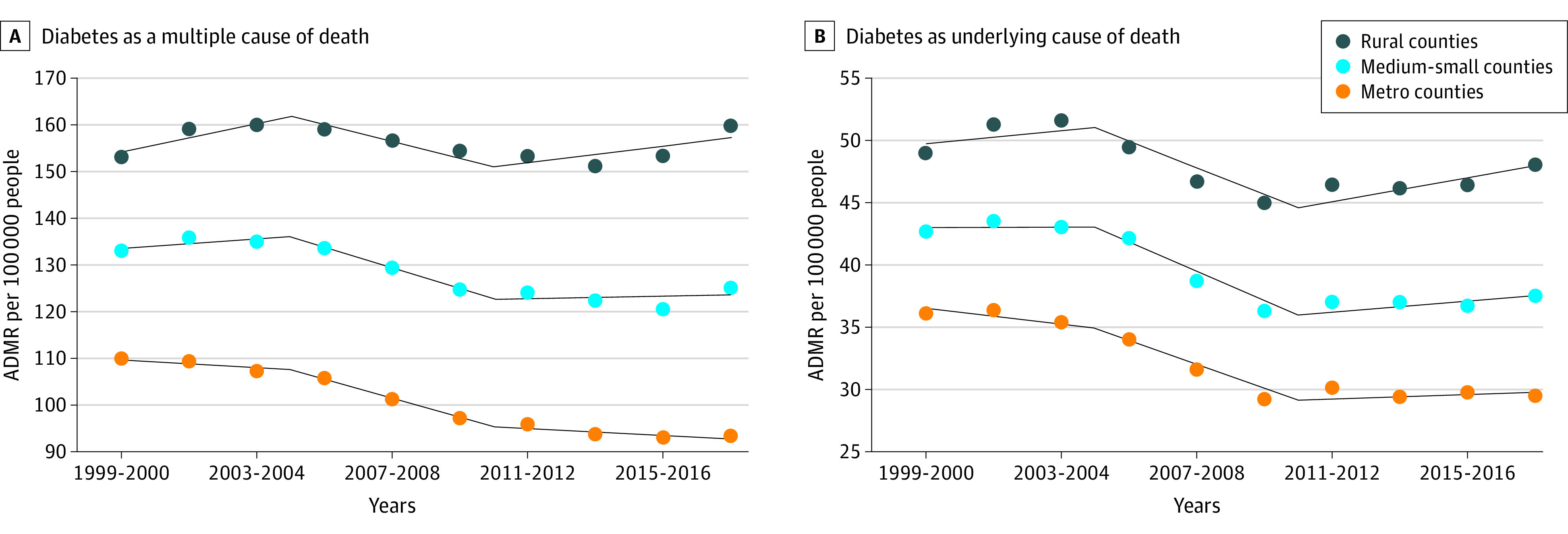
Annual Diabetes Mortality Rate (ADMR) per 100 000 People for Diabetes as Multiple Cause of Death and Underlying Cause of Death Unadjusted estimates included county urbanization, year, 2-way interaction term (county urbanization and year), and age standardized to the 2009-2010 population. Change points occurred at 2003-2004 and 2009-2010. Counties were classified as metro (large central metropolitan and large fringe metropolitan), medium-small (medium metropolitan and small metropolitan), and rural (micropolitan and noncore).

In the overall population, for diabetes as a multiple cause of death, the ADMR was highest in rural compared with medium-small and metro counties (Figure 1; eTable 2 in the Supplement). The ADMR in rural counties was unchanged (157.2 [95% CI, 150.7-163.7] in 2017-2018 vs 154.1 [95% CI, 148.2-160.1] in 1999-2000; *P* = .49) but significantly lower in medium-small counties (123.6 [95% CI, 119.6-127.6] in 2017-2018 vs 133.6 [95% CI, 128.4-138.8] in 1999-2000; *P* = .003) and metro counties (92.9 [95% CI, 90.5-95.3] in 2017-2018 vs 109.7 [95% CI, 105.2-114.1] in 1999-2000; *P* < .001).

### Gender-Specific Diabetes Mortality by County Urbanization

**Figure 2.**
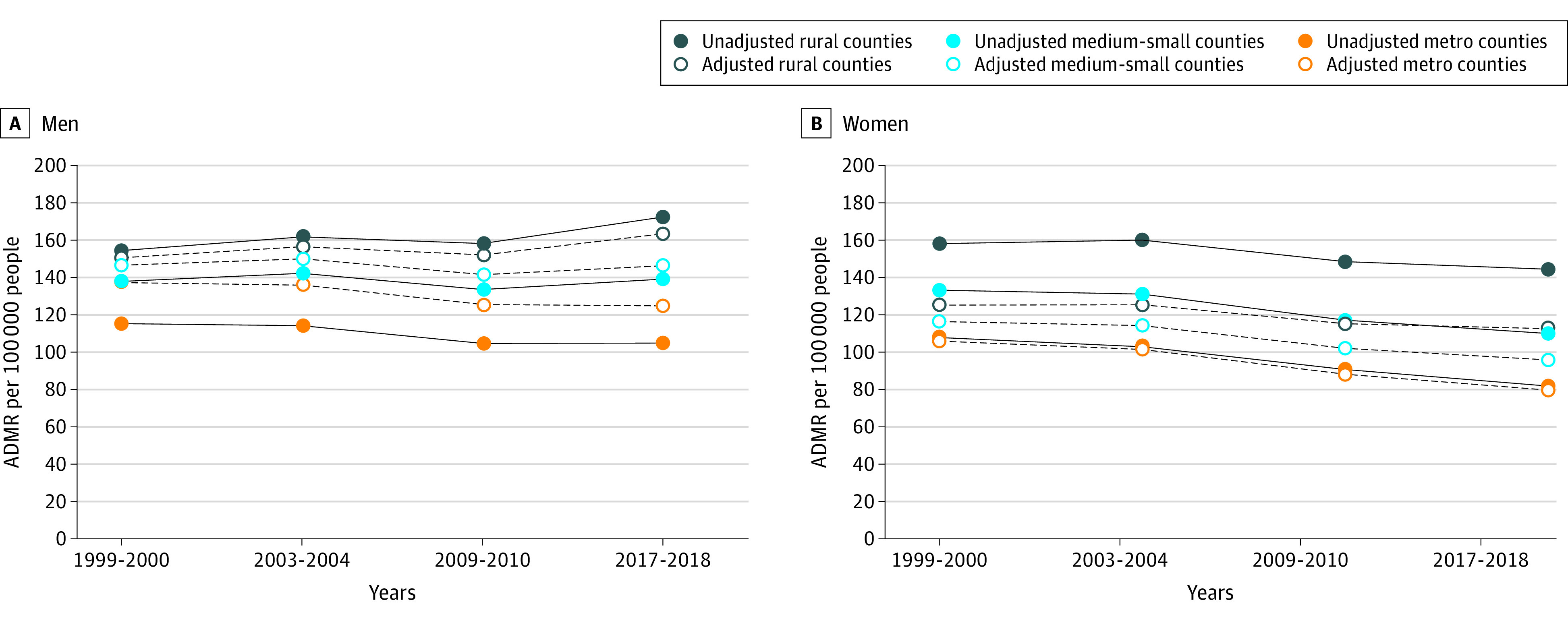
Estimated Annual Diabetes Mortality Rate (ADMR) per 100 000 People by Gender for Diabetes as a Multiple Cause of Death Unadjusted and adjusted estimates included age group, region, up to 3-way interaction terms (gender, county urbanization, and year), and age standardized to the 2009-2010 population for rural, medium-small, and metro counties. Unadjusted estimates represent the effect of the tabled variable, while allowing nontabled variables to vary, as occurring in the original data set. Adjusted estimates represent the hypothetical effects of the same tabled variables if the nontabled variables were equally distributed within each level of the tabled variables (eMethods 2 in the Supplement). The ADMRs and change in ADMRs by county urbanization are listed in the Table and eTable 3 in the Supplement.

In unadjusted estimates for diabetes as a multiple cause of death, the ADMR was significantly higher for men in rural compared with medium-small and metro counties in all years examined (Table; Figure 2; eTable 3 in the Supplement). In 2017-2018 vs 1999-2000, the unadjusted ADMR was significantly higher for men in rural counties (+18.2; 95% CI, 14.3-22.1; *P* < .001) but was unchanged in medium-small counties (+0.7; 95% CI, −1.8 to 3.3) and significantly lower in metro counties (−10.3; 95% CI, −11.9 to −8.7; *P* < .001). In 2017-2018 vs 2009-2010, the ADMR was significantly higher for men in rural counties (+14.2; 95% CI, 10.1-18.3; *P* < .001) and medium-small counties (+5.8; 95% CI, 3.4-8.1; *P* < .001) but was unchanged in metro counties (0.0; 95% CI, −1.6 to 1.6).

**Table.** Estimated Annual Diabetes Mortality Rates for Diabetes as a Multiple Cause of Death

Exposure	Estimated annual diabetes mortality rate per 100 000 people (95% CI)^a^			
	2017-2018 vs 1999-2000		2017-2018 vs 2009-2010	
	Unadjusted estimate	Adjusted estimate	Unadjusted estimate	Adjusted estimate
**Gender**				
Men				
Metro	−10.3 (−11.9 to −8.7)^b^	−12.6 (−14.6 to −10.6)^b^	0.0 (−1.6 to 1.6)	−0.5 (−2.5 to 1.4)
Medium-small	+0.7 (−1.8 to 3.3)	−0.9 (−3.5 to 1.6)	+5.8 (3.4 to 8.1)^b^	+4.7 (2.3 to 7.2)^b^
Rural	+18.2 (14.3 to 22.1)^b^	+12.9 (9.4 to 16.4)^b^	+14.2 (10.1 to 18.3)^b^	+11.2 (7.6 to 14.8)^b^
Women				
Metro	−26.1 (−27.8 to −24.4)^b^	−26.7 (−28.4 to −25.1)^b^	−8.5 (−10.1 to −7.0)^b^	−8.4 (−9.8 to −6.9)^b^
Medium-small	−23.3 (−25.4 to −21.1)^b^	−20.9 (−22.7 to −19.1)^b^	−7.4 (−9.4 to −5.5)^b^	−6.2 (−7.8 to −4.6)^b^
Rural	−14.0 (−17.7 to −10.3)^b^	−12.9 (−15.6 to −10.2)^b^	−4.3 (−7.9 to −0.7)^c^	−2.9 (−5.4 to −0.4)^c^
**Age group, y**				
25-54				
Metro	+0.9 (0.4 to 1.4)^b^	+0.9 (0.4 to 1.4)^b^	+0.1 (−0.2 to 0.4)	+0.1 (−0.2 to 0.4)
Medium-small	+4.5 (4.0 to 5.0)^b^	+4.2 (3.7 to 4.7)^b^	+1.7 (1.3 to 2.1)^b^	+1.6 (1.2 to 2.0)^b^
Rural	+9.4 (8.6 to 10.2)^b^	+8.4 (7.6 to 9.2)^b^	+4.1 (3.4 to 4.8)^b^	+3.6 (2.9 to 4.3)^b^
55-74				
Metro	−46.4 (−48.9 to −43.8)^b^	−46.2 (−48.7 to −43.7)^b^	−1.3 (−3.4 to 0.8)	−0.8 (−3.0 to 1.3)
Medium-small	−27.7 (−31.7 to −23.7)^b^	−29.0 (−32.9 to −25.1)^b^	+7.7 (4.9 to 10.6)^b^	+7.5 (4.8 to 10.3)^b^
Rural	−1.8 (−7.5 to 3.9)	−7.1 (−12.4 to −1.8)^d^	+19.5 (14.8 to 24.2)^b^	+18.0 (13.7 to 22.2)^b^
≥75				
Metro	−73.4 (−85.4 to −61.3)^b^	−77.4 (−89.9 to −64.8)^b^	−45.2 (−56.8 to −33.5)^b^	−45.9 (−57.7 to −34.2)^b^
Medium-small	−55.6 (−70.3 to −40.9)^b^	−55.9 (−70.5 to −41.3)^b^	−40.8 (−53.4 to −28.2)^b^	−40.9 (−53.3 to −28.4)^b^
Rural	−16.8 (−40.5 to 6.8)	−33.0 (−55.5 to −10.5)^d^	−31.1 (−51.9 to −10.2)^d^	−34.4 (−53.1 to −15.6)^b^
**Region**				
Northeast				
Metro	−23.9 (−26.3 to −21.5)^b^	−23.6 (−25.9 to −21.2)^b^	−7.4 (−9.9 to −4.8)^b^	−7.0 (−9.5 to −4.6)^b^
Medium-small	−37.4 (−40.7 to −34.1)^b^	−34.0 (−37.0 to −31.0)^b^	−11.3 (−13.6 to −9.0)^b^	−9.9 (−11.9 to −7.9)^b^
Rural	−37.5 (−42.3 to −32.7)^b^	−31.6 (−35.6 to −27.5)^b^	−12.5 (−16.6 to −8.4)^b^	−10.3 (−13.8 to −6.8)^b^
Midwest				
Metro	−27.4 (−29.1 to −25.7)^b^	−28.7 (−30.5 to −26.9)^b^	−11.0 (−12.5 to −9.5)^b^	−11.5 (−13.1 to −10.0)^b^
Medium-small	−12.2 (−15.2 to −9.3)^b^	−11.7 (−14.5 to −8.9)^b^	−3.6 (−6.6 to −0.5)^c^	−3.2 (−6.1 to −0.3)^c^
Rural	+5.3 (−0.2 to 10.7)	+5.0 (0.5 to 9.4)^c^	+5.8 (−0.5 to 12.1)	+5.6 (0.5 to 10.8)^c^
South				
Metro	−16.0 (−18.4 to −13.6)^b^	−18.1 (−20.8 to −15.4)^b^	−1.1 (−3.3 to 1.1)	−1.5 (−4.0 to 1.0)
Medium-small	−4.8 (−8.6 to −1.1)^d^	−4.2 (−7.7 to −0.6)^c^	+3.3 (−0.3 to 6.9)	+3.3 (−0.1 to 6.6)
Rural	+13.8 (7.6 to 20.0)^b^	+13.3 (7.7 to 18.9)^b^	+10.2 (3.7 to 16.7)^d^	+9.4 (3.6 to 15.3)^d^
West				
Metro	−9.3 (−12.0 to −6.5)^b^	−10.2 (−13.4 to −7.1)^b^	−0.8 (−4.1 to 2.5)	−0.9 (−4.7 to 2.8)
Medium-small	−1.5 (−4.3 to 1.4)	−1.3 (−4.3 to 1.6)	+2.0 (−0.7 to 4.6)	+1.8 (−0.8 to 4.5)
Rural	−0.5 (−4.0 to 3.1)	0.0 (−3.3 to 3.3)	+5.5 (1.8 to 9.2)^d^	+4.6 (1.3 to 8.0)^d^

^a^ Unadjusted and adjusted estimates included the exposure (gender, age group, or region), up to 3-way interaction terms (exposure [gender, age group, or region], county urbanization, or year), and age standardized to the 2009-2010 population (only for gender and region). Estimates are reported in eTable 3 (gender), eTable 4 (age group), and eTable 5 (region) in the Supplement.

^b^ Statistically significant at *P* < .001.

^c^ Statistically significant at *P* < .05.

^d^ Statistically significant at *P* < .01.

The ADMR was significantly higher for women in rural compared with medium-small and metro counties in all years examined (Table; Figure 2; eTable 3 in the Supplement). Overall, in 2017-2018 vs 1999-2000, the ADMR was significantly lower for women at all urbanization levels albeit with the smallest magnitude of decrease in rural counties (−14.0; 95% CI, −17.7 to −10.3; *P* < .001) compared with medium-small counties (−23.3; 95% CI, −25.4 to −21.1; *P* < .001) and metro counties (−26.1; 95% CI, −27.8 to −24.4; *P* < .001). A similar pattern was observed for mortality rates in 2017-2018 vs 2009-2010 in rural counties (−4.3; 95% CI, −7.9 to −0.7; *P* = .01) compared with medium-small counties (−7.4 [−9.4 to −5.5]; *P* < .001) and metro counties (−8.5; 95% CI, −10.1 to −7.0; *P* < .001).

### Age Group–Specific Diabetes Mortality by County Urbanization

**Figure 3.**
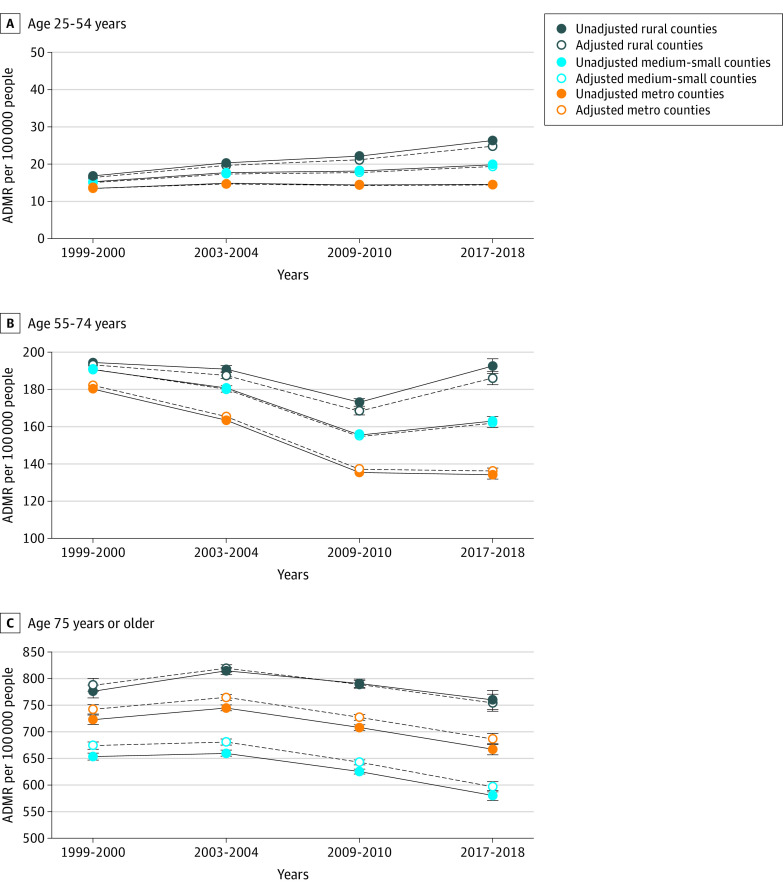
Estimated Annual Diabetes Mortality Rate (ADMR) per 100 000 People by Age Group for Diabetes as a Multiple Cause of Death Unadjusted and adjusted estimates included gender, region, and up to 3-way interaction terms (age group, county urbanization, and year) for rural, medium-small, and metro counties. Note the different y-axis scales. Unadjusted estimates represent the effect of the tabled variable, while allowing nontabled variables to vary, as occurring in the original data set. Adjusted estimates represent the hypothetical effects of the same tabled variables if the nontabled variables were equally distributed within each level of the tabled variables (eMethods 2 in the Supplement). The ADMRs and change in ADMRs by county urbanization are listed in the Table and eTable 4 in the Supplement.

In unadjusted estimates for diabetes as a multiple cause of death, the ADMR was significantly higher in older age groups within all urbanization levels (Table and Figure 3; eTable 4 in the Supplement). In 2017-2018 vs 1999-2000, the ADMR in the 25- to 54-year age group was significantly higher within all urbanization levels, with the largest magnitude of increase in rural counties (+9.4; 95% CI, 8.6-10.2) compared with medium-small counties (+4.5; 95% CI, 4.0-5.0) and metro counties (+0.9; 95% CI, 0.4-1.4) (*P* < .001 for both). In contrast, in the 55- to 74-year age group, the ADMR in 2017-2018 vs 1999-2000 was unchanged in rural counties (−1.8; 95% CI, −7.5 to 3.9) but significantly lower in medium-small counties (−27.7; 95% CI −31.7 to −23.7; *P* < .001) and metro counties (−46.4; 95% CI, −48.9 to −43.8; *P* < .001). Similar to the 55- to 74-year age group, the ADMR in the 75-year or older age group in 2017-2018 vs 1999-2000 was unchanged in rural counties (−16.8; 95% CI, −40.5 to 6.8) but decreased in medium-small counties (−55.6; 95% CI, −70.3 to −40.9; *P* < .001) and metro counties (−73.4; 95% CI, −85.4 to −61.3; *P* < .001).

### Region-Specific Diabetes Mortality by County Urbanization

**Figure 4.**
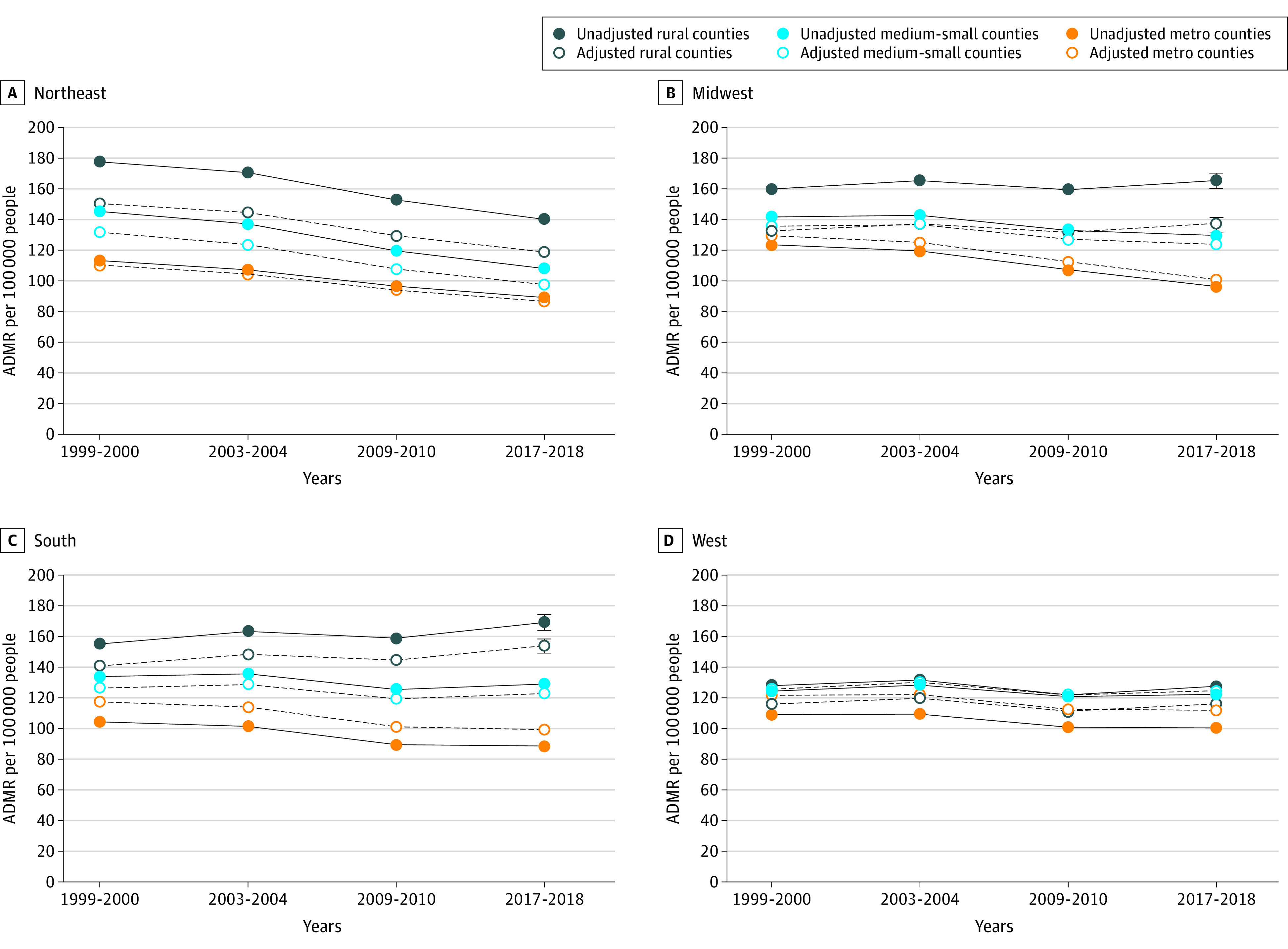
Estimated Annual Diabetes Mortality Rate (ADMR) per 100 000 People by Region for Diabetes as a Multiple Cause of Death Unadjusted and adjusted estimates included gender, age group, up to 3-way interaction terms (region, county urbanization, and year), and age standardized to 2009-2010 population for rural, medium-small, and metro counties. Unadjusted estimates represent the effect of the tabled variable, while allowing nontabled variables to vary, as occurring in the original data set. Adjusted estimates represent the hypothetical effects of the same tabled variables if the nontabled variables were equally distributed within each level of the tabled variables (eMethods 2 in the Supplement). The ADMRs and change in ADMRs by county urbanization are listed in the Table and eTable 5 in the Supplement.

In unadjusted estimates for diabetes as a multiple cause of death, the ADMR in 2017-2018 vs 1999-2000 varied by region (Table and Figure 4; eTable 5 in the Supplement). In the Northeast, the improvement in mortality rate was significantly higher in rural counties (−37.5; 95% CI, −42.3 to −32.7; *P* < .001) compared with metro counties (−23.9; 95% CI, −26.3 to −21.5; *P* < .001) but similar to medium-small counties (−37.4; 95% CI, −40.7 to −34.1; *P* < .001). In contrast, the ADMR was unchanged in the rural Midwest (+5.3; 95% CI, −0.2 to 10.7) compared with the metro Midwest (−27.4; 95% CI, −29.1 to −25.7; *P* < .001) and in the rural West (−0.5; 95% CI, −4.0 to 3.1) compared with the metro West (−9.3; 95% CI, −12.0 to −6.5; *P* < .001). The rural disparity was pronounced in the South: the ADMR in 2017-2018 vs 1999-2000 was significantly higher in rural counties (+13.8; 95% CI, 7.6-20.0; *P* < .001) but lower in medium-small counties (−4.8; 95% CI, −8.6 to −1.1; *P* = .01) and metro counties (−16.0; 95% CI, −18.4 to −13.6; *P* < .001). Similar trends were generally observed for 2017-2018 vs 2009-2010.

### Adjusted Estimates

For gender, age group, and region, the ADMR levels were less variable across rurality after adjustment (Table and Figures 2-4; eTables 3-5 in the Supplement). For instance, in 1999-2000 for men, the ADMR before adjustment was 115.3 (95% CI, 114.3-116.3) in metro counties and 154.3 (95% CI, 152.2-156.5) in rural counties; after adjustment, the ADMR was 137.4 (95% CI, 136.2-138.7) in metro counties and 150.6 (95% CI, 148.6-152.5) in rural counties, suggesting that adjustment for age group and region partially accounted for the differences in ADMR level across rurality levels. Similarly, in 1999-2000 for women, the ADMR before adjustment was 108.0 (95% CI, 106.8-109.2) in metro counties and 158.3 (95% CI, 155.7-160.9) in rural counties and 106.4 (95% CI, 105.2-107.7) in metro counties and 125.3 (95% CI, 123.4-127.3) in rural counties after adjustment, suggesting that adjustment for age group and region partially accounted for the differences in level.

In contrast, variation across rurality in the ADMR differences (2017-2018 vs 1999-2000) was generally similar in unadjusted vs adjusted estimates. For men, the ADMR in 2017-2018 vs 1999-2000 in unadjusted estimates was +18.2 (95% CI, 14.3-22.1) in rural counties and −10.3 (95% CI, −11.9 to −8.7) in metro counties (difference of changes in ADMR, 28.5); the ADMR in adjusted estimates was +12.9 (95% CI, 9.4-16.4) in rural counties and −12.6 (95% CI, −14.6 to −10.6) in metro counties (difference of changes in ADMR, 25.5). Similarly, for women, the ADMR difference between metro and rural counties (2017-2018 vs 1999-2000) was generally similar at +12.1 (unadjusted estimate) and +13.8 (adjusted estimate).

### Temporal Trends in Mortality and Exposures

We examined whether the variation in mortality time trends with respect to other exposures (ie, gender, age group, and region) accounted for the variation in mortality time trends across county urbanization levels. The F statistic for the interactions between time and urbanization was 34.0 before and 31.1 after inclusion of other time × exposure interactions. This finding indicated that almost all variation in ADMR time trends attributable to county urbanization was independent of variation in ADMR time trends attributable to other exposures.

### Diabetes as Underlying Cause of Death

Compared with the multiple cause of death analysis, the absolute ADMR for diabetes as the underlying cause of death was lower. Results observed in the multiple cause of death analysis were generally preserved (Figure 1; eTables 6-8 in the Supplement).

## Discussion

In this analysis, the ADMR in 2017-2018 vs 1999-2000 was lower in women at all urbanization levels, albeit with the smallest improvement in mortality in rural counties. In contrast, the ADMR in men was higher in rural counties but unchanged or lower in other counties. In the 25- to 54-year age group, the ADMR was higher at all urbanization levels, with the highest mortality rate in rural counties. In contrast, ADMRs in the 55- to 74-year and 75-year or older age groups were lower in medium-small and metro counties but unchanged in rural counties. When stratified by region, the ADMR in 2017-2018 vs 1999-2000 was higher in the rural South, unchanged in the rural Midwest and rural West, and lower in the rural Northeast. Similar results were generally obtained for the ADMR in 2017-2018 vs 2009-2010. Taken together, the results describe subnational trends in diabetes mortality, highlight persistent rural disparities, and identify areas for investigation and intervention.

In the US, from 1990 to 2017, the increase in the annual age-adjusted prevalence of diabetes from approximately 1990 through 2009-2010 for men and women was attributed in part to an increase in incidence and a decrease in mortality.^4,10^ Although the national mortality trend was reassuring, subnational results in the current study show that the decrease in mortality was driven by nonrural counties; the ADMR increased in rural men and improved the least in rural women compared with their nonrural counterparts. Previous studies^4,10,11^ described the gender differences in trends for diabetes prevalence, incidence, and mortality. However, the determinants of gender differences in mortality in rural vs nonrural counties are unknown and may be linked to differences in diabetes care, adherence to therapy, and incidence of diabetes complications, among other factors.

The US annual prevalence of diabetes for adults aged 65 to 79 years increased from 1990 through approximately 2010 and then plateaued.^4^ Since 2011, the annual incidence of diabetes has decreased by 8.1%.^4^ Along with a decrease in incidence, results from the current study showed that ADMR decreased in adults 75 years or older and accounted for the plateau in diabetes prevalence. In the current study, the decrease in ADMR in adults 75 years or older was nonuniform across urbanization levels, with a small improvement in rural counties. Although the reasons are incompletely understood, a cross-sectional study^12^ of US adults 65 years or older with diabetes showed that rural, compared with nonrural, residents had higher odds of not having a health care practitioner, deferring care because of medical cost, and having an annual household income of less than $35 000, placing them at greater risk of not receiving adequate diabetes care. The reasons for the increasing ADMR in the 25- to 54-year age group are unclear and may include a higher proportion of uninsured adults, lower family income, and lower wages, among other factors.^13^

In the current study, the trend in the ADMR for diabetes as a multiple cause of death differed by region: in unadjusted estimates in 2017-2018 vs 1999-2000, the ADMR was higher in the rural South and unchanged or lower in other rural regions. Regional differences in the ADMR may be linked to differences in disease burden, with a higher age-adjusted prevalence of diabetes in the South,^14,15^ modifiable risk factors (eg, physical activity),^16^ environmental pollution,^17,18,19,20,21^ and race or ethnicity, among other factors.^22^ Interventions specific to the rural South have examined the role of education programs and technology to improve diabetes care,^23,24^ and studies are required to evaluate their generalizability to other regions.

In the current study, the ADMR for diabetes as a multiple cause of death decreased in rural and nonrural counties, but rural disparities persisted independent of variation in ADMR time trends attributable to gender, age group, and region. These rural disparities may be linked to differences in diabetes comorbidities,^25^ consumption of fruits and vegetables,^26,27^ environmental toxins,^28^ suboptimal diabetes management,^29,30,31^ health care access (eg, for dilated eye examination),^32,33^ and change in treatment targets,^34^ which are major challenges in rural counties. For instance, diabetes is a major cardiovascular risk factor,^35,36,37^ and despite overall improvement in cardiovascular mortality in rural and nonrural counties, rural-nonrural disparities persist.^8^ Addressing systemic rural-nonrural differences may improve outcomes for diabetes and other conditions.

Diabetes is a major public health problem, and the higher rural prevalence has prompted evaluation and implementation of rural-specific technology interventions,^38^ community programs,^39,40^ and redesigned primary care models.^41^ These programs focus on improving glycemic control, lipid levels, and blood pressure, and additional studies are required to assess whether they reduce the incidence of diabetes complications (eg, cardiovascular disease) and mortality. The rural-nonrural disparities prompted the American Heart Association and American Stroke Association to release a 2020 Call to Action advisory to improve rural health.^42^

### Strengths and Limitations

This study has several strengths. A previous study^1^ showed that diabetes mortality rates were lagging in rural areas. In the current study, we used a nonbiased approach to identify change points and evaluate trends. To our knowledge, the current study is the most comprehensive analysis of subnational mortality trends based on gender, age group, region, and rurality (and interactions) using unadjusted and adjusted estimates for diabetes as a multiple cause and the underlying cause of death. This study showed that gender, age group, region, and additional factors contribute to rural-nonrural differences in mortality.

This study also has some limitations. The analysis used data from death certificates, which may not always list diabetes as a contributor to mortality. However, we do not have evidence of nonrandom reporting of diabetes as a contributor to mortality. To mitigate potential nonreporting of diabetes on death certificates, we analyzed diabetes as a multiple cause of death and the underlying cause.^43,44^ Death certificates include type 1 and type 2 diabetes. However, approximately 95% of diabetes cases are type 2 diabetes in adults older than 25 years, and the results are likely representative of type 2 diabetes. We did not stratify by race or ethnicity to avoid suppressed data and erroneous estimates; the association between race or ethnicity and diabetes mortality has been reported.^44^ We focused on condensed urbanization levels and did not have corresponding data (eg, health insurance) to explain the rurality-mortality association.

## Conclusions

In this analysis of diabetes mortality from 1999 to 2018, rural counties demonstrated higher mortality rates compared with other urbanization levels. Subnational disparities were found based on gender, age group, and region. For diabetes as a multiple cause of death, trends in mortality rates for rural men, adults aged 25 to 54 years, and adults in the rural South and rural Midwest were most concerning. The variation in ADMR trends by county urbanization was independent of variation in the ADMR time trends attributable to gender, age group, and region, suggesting the contribution of additional factors.

Our results highlight the importance of exploring individual and community factors that determine mortality in rural counties. This knowledge will inform further investigation into the determinants of mortality and targeted interventions to close the rural-nonrural gap and improve rural health, which is a priority of the Healthy People 2030 agenda.^45^

## Notes

Supplement. **eMethods 1.** Modeling Diabetes Mortality Rates **eMethods 2. **Unadjusted and Adjusted Estimates for Diabetes Mortality **eMethods 3.** Comparison of Simple and Full Models **eTable 1.** United States Population Characteristics From 1999–2018, by County Urbanization **eTable 2.** Change in Estimated Annual Diabetes Mortality Rates (ADMR), 1999–2018, by Year and County Urbanization **eTable 3.** Estimated Annual Diabetes Mortality Rates (ADMR), 1999–2018, by Sex and County Urbanization, for Diabetes as a Multiple Cause of Death **eTable 4.** Estimated Annual Diabetes Mortality Rates (ADMR), 1999–2018, by Age Group and County Urbanization, for Diabetes as a Multiple Cause of Death **eTable 5.** Estimated Annual Diabetes Mortality Rates (ADMR), 1999–2018, by Region and County Urbanization, for Diabetes as a Multiple Cause of Death **eTable 6.** Estimated Annual Diabetes Mortality Rates (ADMR), 1999–2018, by Sex and County Urbanization, for Diabetes as the Underlying Cause of Death **eTable 7.** Estimated Annual Diabetes Mortality Rates (ADMR), 1999–2018, by Age Group and County Urbanization, for Diabetes as the Underlying Cause of Death **eTable 8.** Estimated Annual Diabetes Mortality Rates (ADMR), 1999–2018, by Region and County Urbanization, for Diabetes as the Underlying Cause of Death
