# Supplementary material for: Assessment of Disparities in Diabetes Mortality in Adults in US Rural vs Nonrural Counties, 1999-2018
Source: JAMA Netw Open. 2022 Sep 20;5(9):e2232318. doi: 10.1001/jamanetworkopen.2022.32318 (PMC9490502; doi:10.1001/jamanetworkopen.2022.32318)
Supplement: Supplement. — eMethods 1. Modeling Diabetes Mortality Rates eMethods 2. Unadjusted and Adjusted Estimates for Diabetes Mortality eMethods 3. Comparison of Simple and Full Models eTable 1. United States Population Characteristics From 1999–2018, by County Urbanization eTable 2. Change in Estimated Annual Diabetes Mortality Rates (ADMR), 1999–2018, by Year and County Urbanization eTable 3. Estimated Annual Diabetes Mortality Rates (ADMR), 1999–2018, by Sex and County Urbanization, for Diabetes as a Multiple Cause of Death eTable 4. Estimated Annual Diabetes Mortality Rates (ADMR), 1999–2018, by Age Group and County Urbanization, for Diabetes as a Multiple Cause of Death eTable 5. Estimated Annual Diabetes Mortality Rates (ADMR), 1999–2018, by Region and County Urbanization, for Diabetes as a Multiple Cause of Death eTable 6. Estimated Annual Diabetes Mortality Rates (ADMR), 1999–2018, by Sex and County Urbanization, for Diabetes as the Underlying Cause of Death eTable 7. Estimated Annual Diabetes Mortality Rates (ADMR), 1999–2018, by Age Group and County Urbanization, for Diabetes as the Underlying Cause of Death eTable 8. Estimated Annual Diabetes Mortality Rates (ADMR), 1999–2018, by Region and County Urbanization, for Diabetes as the Underlying Cause of Death [file jamanetwopen-e2232318-s001.pdf]

Supplementary Online Content

Dugani SB, Wood-Wentz CM, Mielke MM, Bailey KR, Vella A. Assessment of disparities in diabetes mortality in adults in US rural vs nonrural counties, 1999-2018. *JAMA Netw Open*. 2022;5(9):e2232318. doi:10.1001/jamanetworkopen.2022.32318

eMethods 1. Modeling Diabetes Mortality Rates
eMethods 2. Unadjusted and Adjusted Estimates for Diabetes Mortality
eMethods 3. Comparison of Simple and Full Models
eTable 1. United States Population Characteristics From 1999–2018, by County Urbanization
eTable 2. Change in Estimated Annual Diabetes Mortality Rates (ADMR), 1999–2018, by Year and County Urbanization
eTable 3. Estimated Annual Diabetes Mortality Rates (ADMR), 1999–2018, by Sex and County Urbanization, for Diabetes as a Multiple Cause of Death
eTable 4. Estimated Annual Diabetes Mortality Rates (ADMR), 1999–2018, by Age Group and County Urbanization, for Diabetes as a Multiple Cause of Death
eTable 5. Estimated Annual Diabetes Mortality Rates (ADMR), 1999–2018, by Region and County Urbanization, for Diabetes as a Multiple Cause of Death
eTable 6. Estimated Annual Diabetes Mortality Rates (ADMR), 1999–2018, by Sex and County Urbanization, for Diabetes as the Underlying Cause of Death
eTable 7. Estimated Annual Diabetes Mortality Rates (ADMR), 1999–2018, by Age Group and County Urbanization, for Diabetes as the Underlying Cause of Death
eTable 8. Estimated Annual Diabetes Mortality Rates (ADMR), 1999–2018, by Region and County Urbanization, for Diabetes as the Underlying Cause of Death

This supplementary material has been provided by the authors to give readers additional information about their work.

© 2022 Dugani SB et al. *JAMA Network Open.*

eMethods 1. Modeling Diabetes Mortality Rates

To model time, we considered the following model fits: 2 time segments (1 change point), 3 time segments (2 change points), and 4 time segments (3 change points). Based on the F-statistic, there was a statistically significant improvement in the model fit in going from ‘2 time segments’ to ‘3 time segments’. However, there was no statistically significant improvement in going from ‘3 time segments’ to ‘4 time segments’. Therefore, we used the ‘3 time segments’ fit.

Considering 720 cells (3 urbanization categories × 3 age-groups × 2 sexes × 4 regions × 10 time points) as independent observations, weighted multiple linear regression was used to model the outcome [log (mortality rate)], where mortality was calculated as deaths per population in each cell, and reported as annual diabetes mortality rate per 100,000 people (ADMR). Under the assumption that log (mortality rate) for a given cell was based on the underlying Poisson count variable, the optimal weight for a given data cell would be the expected mortality count for that cell. We used a main effects model (region, county urbanization, age-group, and sex, used as nominal variables, and time as a 3-segment linear function), to estimate optimal weights as predicted mortality counts. The procedure was iterated until it converged, and the resulting weights were used for all subsequent analyses. Although the Poisson assumption was used to provide weights, standard errors of parameter estimates were based on the model, i.e., they used the residual mean squared error (MSE).

Weighted regression was used on the assumption that the 720 data points (counts of diabetes related deaths and population denominators) have a Poisson variation. However, the Poisson assumption may overstate the precision. Therefore, we modeled the log of the rate (counts of diabetes related deaths/population) in a general linear model, maintaining the consequence of the Poisson assumption that the cells have different precision. Therefore, we used weights proportional to the expected counts. This resulted in substantially smaller standard errors than those for unweighted regression.

The model for log (mortality rate) was based on consideration of all main effects and up to 3-way interactions. Interaction terms were pruned based on the statistical significance. The hierarchical structure was maintained such that inclusion of a high-order interaction required inclusion of corresponding lower-order interaction terms.

© 2022 Dugani SB et al. *JAMA Network Open.*

eMethods 2. Unadjusted and Adjusted Estimates for Diabetes Mortality

To determine unadjusted estimates, we added predicted death counts and population denominators for each combination of variables, along with age-group, to obtain mortality rates for table cell (for each age-group). These rates were age-standardized to the 2009–2010 population by taking weighted means across age-groups, using weights determined by the 2009–2010 age distribution within our dataset.

To determine adjusted estimates, we created and appended copies of the original dataset without the Y-variable [log (mortality rate)], one for each cell, such that the non-tabled variables had the distribution of the original dataset with the variables fixed to a particular set of values. These counts were added over the non-tabled variable for each combination of table value and age-group, with the same age adjustment as for the unadjusted tables. The predicted log (mortality rate) was calculated for each combination.

© 2022 Dugani SB et al. *JAMA Network Open.*

eMethods 3. Comparison of Simple and Full Models

To examine if variation in mortality time trends with respect to other exposures (i.e., sex, age-group, and region) accounted for the variation in mortality time trends across urbanization categories, we compared F-statistics from two sets of models: simple and full. The 'simple' set compared a model with no time*exposure interactions with one containing only the time*urbanization interactions (6 numerator d.f.), which had an F-statistic of 34.0. The 'full' set was based on adding time*urbanization interactions to a model with all other time*X interactions (6 numerator d.f.), which had an F-statistic of 31.1. Both models contained all main effects and all 2-way interactions other than those involving time. The same denominator mean square based on 661 d.f. was used in both cases. The similar F-statistic values suggested that almost all variation in time trends due to urbanization was independent of variation in time trends with respect to other exposures.

© 2022 Dugani SB et al. *JAMA Network Open.*

eTable 1. United States Population Characteristics From 1999–2018, by County Urbanization

	County urbanization		
	Metro (%)	Medium-Small (%)	Rural (%)
Women	52.1	51.9	51.0
Age-group, years			
25–54	65.2	61.0	57.1
55–74	26.4	29.2	31.9
≥75	8.5	9.8	11.0
Region			
Midwest	19.1	21.0	33.1
South	32.8	41.3	42.5
West	25.6	22.3	13.9
Northeast	22.5	15.5	10.5

Data represent the mean for metro (large central metro + large fringe metro), medium-small (medium metro + small metro), and rural (micropolitan + non-core) counties.

© 2022 Dugani SB et al. *JAMA Network Open.*

eTable 2. Change in Estimated Annual Diabetes Mortality Rates (ADMR), 1999–2018, by Year and County Urbanization

	Difference in mortality rate, per 100,000 people (95% confidence interval)
Diabetes as multiple cause of death	
2017–2018 vs. 1999–2000	
Metro	-16.8 (-22.0 to -11.7)***
Medium-Small	-10.0 (-16.7 to -3.3)*
Rural	3.0 (-5.9 to 12.0)
2017–2018 vs. 2009–2010	
Metro	-4.4 (-7.7 to -1.2)*
Medium-Small	-1.3 (-6.3 to 3.8)
Rural	4.4 (-3.6 to 12.4)
Diabetes as underlying cause of death	
2017–2018 vs. 1999–2000	
Metro	-6.7 (-9.0 to -4.5)***
Medium-Small	-5.5 (-8.1 to -2.9)***
Rural	-1.8 (-5.5 to 2.0)
2017–2018 vs. 2009–2010	
Metro	-0.3 (-1.8 to 1.2)
Medium-Small	0.5 (-1.8 to 2.7)
Rural	2.4 (-1.2 to 5.9)

Estimated annual diabetes mortality rates (ADMR [95% confidence interval]; per 100,000 people) from models that included county urbanization, year, 2-way interaction (county urbanization, year), and age-standardized to the 2009–2010 population.
Statistical significance at [a]P<.05; *P<.01; **P<.001; ***P<.0001

© 2022 Dugani SB et al. *JAMA Network Open.*

eTable 3. Estimated Annual Diabetes Mortality Rates (ADMR), 1999–2018, by Sex and County Urbanization, for Diabetes as a Multiple Cause of Death

	**Estimated annual diabetes mortality rate, per 100,000 people**							
	Unadjusted estimates				Adjusted estimates			
Sex	1999-2000	2003-2004	2009-2010	2017-2018	1999-2000	2003-2004	2009-2010	2017-2018
Men								
Metro	115.3*** (114.3-116.3)	114.1 (113.4-114.8)	104.9 (104.3-105.6)	104.9 (103.7-106.2)	137.4*** (136.2-138.7)	136.1 (135.3-136.9)	125.4 (124.6-126.2)	124.8 (123.4-126.3)
Medium-Small	138.6 (137.0-140.3)	141.9 (140.9-142.8)	133.6*** (132.7-134.5)	139.4 (137.5-141.3)	147.3 (145.6-148.9)	150.4 (149.5-151.4)	141.6** (140.7-142.6)	146.3 (144.4-148.3)
Rural	154.3*** (152.2-156.5)	161.9 (160.6-163.2)	158.3*** (156.7-159.9)	172.5 (169.2-175.9)	150.6*** (148.6-152.5)	156.9 (155.7-158.1)	152.2*** (150.8-153.7)	163.4 (160.6-166.3)
Women								
Metro	108.0*** (106.8-109.2)	102.8 (102.1-103.6)	90.5*** (89.8-91.1)	81.9 (80.8-83.1)	106.4*** (105.2-107.7)	100.9 (100.2-101.7)	88.1*** (87.5-88.7)	79.7 (78.6-80.8)
Medium-Small	133.0*** (131.4-134.6)	130.8 (129.9-131.8)	117.2*** (116.3-118.1)	109.7 (108.3-111.2)	116.6*** (115.2-117.9)	114.2 (113.3-115.0)	101.8*** (101.1-102.6)	95.6 (94.5-96.8)
Rural	158.3*** (155.7-160.9)	160.0 (158.6-161.5)	148.5[a] (146.9-150.2)	144.3 (141.5-147.0)	125.3*** (123.4-127.3)	125.4 (124.4-126.5)	115.3[a] (114.2-116.5)	112.4 (110.4-114.4)

Estimated annual diabetes mortality rates (ADMR [95% confidence interval]; per 100,000 people) from unadjusted and adjusted estimates that included age-group, region, up to 3-way interaction terms (sex, county urbanization, year), and age-standardized to the 2009–2010 population.
Statistical significance at [a]P<.05; *P<.01; **P<.001; ***P<.0001 compared to 2017–2018.

© 2022 Dugani SB et al. *JAMA Network Open.*

eTable 4. Estimated Annual Diabetes Mortality Rates (ADMR), 1999–2018, by Age Group and County Urbanization, for Diabetes as a Multiple Cause of Death

	**Estimated annual diabetes mortality rate, per 100,000 people**							
	Unadjusted estimates				Adjusted estimates			
Age-group, years	1999-2000	2003-2004	2009-2010	2017-2018	1999-2000	2003-2004	2009-2010	2017-2018
25–54								
Metro	13.5** (13.1-13.8)	14.7 (14.6-14.9)	14.3 (14.1-14.4)	14.4 (14.1-14.6)	13.5** (13.1-13.9)	14.8 (14.6-14.9)	14.3 (14.2-14.4)	14.4 (14.2-14.6)
Medium-Small	15.3*** (15.0-15.7)	17.7 (17.5-17.9)	18.1*** (17.9-18.3)	19.8 (19.5-20.1)	15.1*** (14.7-15.4)	17.3 (17.1-17.6)	17.7*** (17.5-17.9)	19.3 (19.0-19.6)
Rural	16.9*** (16.5-17.3)	20.3 (19.9-20.6)	22.1*** (21.8-22.5)	26.3 (25.6-26.9)	16.4*** (16.0-16.8)	19.6 (19.3-19.9)	21.2*** (20.9-21.5)	24.8 (24.2-25.4)
55–74								
Metro	180.4*** (178.6-182.2)	163.4 (162.4-164.5)	135.3 (134.3-136.4)	134.0 (132.4-135.7)	182.3*** (180.6-184.1)	165.3 (164.3-166.4)	137.0 (135.9-138.0)	136.1 (134.4-137.8)
Medium-Small	190.7*** (187.7-193.7)	180.6 (179.1-182.1)	155.3*** (154.1-156.5)	163.0 (160.7-165.4)	190.9*** (188.0-193.8)	180.0 (178.5-181.4)	154.3*** (153.2-155.5)	161.8 (159.6-164.1)
Rural	194.4 (190.8-198.0)	191.0 (189.1-192.8)	173.1*** (171.0-175.1)	192.6 (188.6-196.5)	193.2* (189.8-196.5)	187.7 (185.9-189.5)	168.1*** (166.3-169.9)	186.1 (182.5-189.6)
≥75								
Metro	653.6*** (646.9-660.3)	659.8 (654.3-665.2)	625.4*** (620.9-629.9)	580.2 (571.0-589.5)	674.1*** (667.1-681.2)	680.8 (675.2-686.3)	642.7*** (638.5-646.9)	596.8 (587.4-606.1)
Medium-Small	722.9*** (714.1-731.7)	745.0 (739.8-750.3)	708.1*** (702.9-713.3)	667.3 (656.6-678.0)	742.2*** (733.4-751.1)	764.9 (759.6-770.3)	727.2*** (722.0-732.4)	686.3 (675.8-696.9)
Rural	776.6 (763.6-789.7)	814.7 (807.6-821.8)	790.9* (782.7-799.1)	759.8 (742.1-777.6)	787.7* (774.9-800.6)	819.6 (812.7-826.5)	789.1** (781.6-796.7)	754.7 (738.6-770.9)

Estimated annual diabetes mortality rates (ADMR [95% confidence interval]; per 100,000 people) from unadjusted and adjusted estimates that included sex, region, and up to 3-way interaction terms (age-group, county urbanization, year).
Statistical significance at *P<.01; **P<.001; ***P<.0001 compared to 2017–2018.

© 2022 Dugani SB et al. *JAMA Network Open.*

eTable 5. Estimated Annual Diabetes Mortality Rates (ADMR), 1999–2018, by Region and County Urbanization, for Diabetes as a Multiple Cause of Death

	Estimated annual diabetes mortality rate, per 100,000 people							
	Unadjusted estimates				Adjusted estimates			
Region	1999-2000	2003-2004	2009-2010	2017-2018	1999-2000	2003-2004	2009-2010	2017-2018
Northeast								
Metro	113.2*** (111.5-114.9)	107.2 (106.3-108.1)	96.6*** (95.4-97.9)	89.3 (87.5-91.0)	110.3*** (108.7-112.0)	104.3 (103.4-105.1)	93.7*** (92.5-95.0)	86.7 (85.0-88.4)
Medium-Small	145.8*** (142.8-148.7)	137.4 (135.9-138.8)	119.7*** (118.5-120.9)	108.4 (106.7-110.1)	131.6*** (129.0-134.2)	123.8 (122.5-125.1)	107.5*** (106.5-108.6)	97.6 (96.1-99.2)
Rural	177.8*** (174.2-181.4)	170.9 (168.9-172.9)	152.8*** (150.8-154.8)	140.3 (137.0-143.7)	150.6*** (147.6-153.7)	144.8 (143.1-146.4)	129.4*** (127.7-131.1)	119.1 (116.3-121.9)
Midwest								
Metro	123.7*** (122.4-125.0)	119.5 (118.6-120.3)	107.3*** (106.7-108.0)	96.4 (95.3-97.5)	129.5*** (128.2-130.9)	125.1 (124.2-126.0)	112.3*** (111.6-113.0)	100.8 (99.7-102.0)
Medium-Small	141.7*** (139.6-143.8)	142.8 (141.3-144.2)	133.0[a] (131.5-134.5)	129.5 (127.3-131.6)	135.5*** (133.5-137.6)	136.4 (135.1-137.8)	127.0[a] (125.5-128.4)	123.8 (121.8-125.9)
Rural	160.0 (157.6-162.4)	165.4 (164.0-166.8)	159.5 (157.3-161.6)	165.3 (160.2-170.3)	132.3[a] (130.3-134.3)	136.6 (135.5-137.8)	131.6[a] (129.8-133.4)	137.2 (133.1-141.3)
South								
Metro	104.5*** (102.8-106.1)	101.2 (100.3-102.2)	89.5 (88.9-90.2)	88.5 (86.7-90.2)	117.4*** (115.6-119.3)	113.8 (112.7-114.9)	100.9 (100.1-101.6)	99.3 (97.4-101.3)
Medium-Small	133.7** (131.4-136.0)	135.8 (134.8-136.8)	125.6 (124.5-126.7)	128.9 (125.9-131.8)	126.7[a] (124.5-128.9)	128.8 (127.8-129.8)	119.3 (118.2-120.3)	122.5 (119.8-125.3)
Rural	155.6*** (151.7-159.4)	163.6 (161.7-165.5)	159.1* (156.7-161.6)	169.3 (164.3-174.4)	140.7*** (137.2-144.2)	148.4 (146.6-150.1)	144.6* (142.3-146.8)	154.0 (149.4-158.6)
West								
Metro	109.2*** (107.9-110.6)	109.5 (108.2-110.8)	100.7 (99.4-102.1)	100.0 (97.5-102.4)	121.9*** (120.4-123.4)	122.2 (120.7-123.7)	112.6 (111.0-114.2)	111.7 (108.9-114.5)
Medium-Small	124.0 (121.7-126.3)	128.3 (126.8-129.8)	120.5 (119.2-121.9)	122.5 (120.7-124.3)	125.2 (122.9-127.5)	129.7 (128.1-131.2)	122.0 (120.6-123.4)	123.8 (122.0-125.7)
Rural	127.8 (125.6-130.0)	131.4 (129.7-133.0)	121.9* (120.2-123.5)	127.4 (124.5-130.2)	116.0 (114.0-118.0)	119.7 (118.2-121.2)	111.4* (109.8-112.9)	116.0 (113.4-118.7)

Estimated annual diabetes mortality rates (ADMR [95% confidence interval]; per 100,000 people) from unadjusted and adjusted estimates that included sex, age-group, up to 3-way interaction terms (region, county urbanization, year), and age-standardized to the 2009–2010 population.
Statistical significance at [a]P<.05; *P<.01; **P<.001; ***P<.0001 compared to 2017–2018.

© 2022 Dugani SB et al. *JAMA Network Open.*

eTable 6. Estimated Annual Diabetes Mortality Rates (ADMR), 1999–2018, by Sex and County Urbanization, for Diabetes as the Underlying Cause of Death

	Estimated annual diabetes mortality rate, per 100,000 people							
	Unadjusted estimates				Adjusted estimates			
Sex	1999-2000	2003-2004	2009-2010	2017-2018	1999-2000	2003-2004	2009-2010	2017-2018
Men								
Metro	37.0*** (36.4-37.6)	36.3 (36.0-36.6)	32.2 (31.8-32.6)	32.2 (31.8-32.6)	43.9*** (43.3-44.5)	43.4 (43.0-43.8)	38.1 (37.6-38.6)	39.9 (39.2-40.6)
Medium-Small	42.9 (42.2-43.6)	43.9 (43.5-44.2)	39.5 (39.1-40.0)	43.1 (42.4-43.7)	44.7 (44.0-45.4)	46.0 (45.6-46.4)	41.3 (40.8-41.8)	44.9 (44.2-45.6)
Rural	47.5*** (46.4-48.6)	49.7 (49.0-50.5)	46.8 (46.1-47.5)	53.2 (52.2-54.2)	45.6*** (44.5-46.6)	48.0 (47.3-48.6)	44.9 (44.2-45.6)	50.8 (49.9-51.7)
Women								
Metro	37.1*** (36.6-37.7)	34.4 (34.1-34.8)	28.1 (27.7-28.5)	26.0 (25.6-26.4)	37.1*** (36.5-37.8)	34.0 (33.7-34.4)	27.6 (27.3-28.0)	25.4 (25.1-25.8)
Medium-Small	44.1*** (43.3-44.8)	42.5 (42.0-42.9)	35.0 (34.5-35.4)	33.1 (32.6-33.5)	38.7*** (38.0-39.4)	36.9 (36.5-37.3)	30.6 (30.2-30.9)	29.2 (28.8-29.7)
Rural	52.8*** (51.6-54.1)	52.2 (51.3-53.0)	44.9 (44.1-45.6)	44.0 (43.0-45.1)	41.8*** (40.8-42.8)	40.8 (40.2-41.5)	35.3 (34.7-35.9)	35.2 (34.5-35.9)

Estimated annual diabetes mortality rates (ADMR [95% confidence interval]; per 100,000 people) from unadjusted and adjusted estimates that included age-group, region, up to 3-way interaction terms (sex, county urbanization, year), and age-standardized to the 2009–2010 population.
Statistical significance at ***P<.0001 compared to 2017–2018.

© 2022 Dugani SB et al. *JAMA Network Open.*

eTable 7. Estimated Annual Diabetes Mortality Rates (ADMR), 1999–2018, by Age Group and County Urbanization, for Diabetes as the Underlying Cause of Death

	**Estimated annual diabetes mortality rate, per 100,000 people**							
	Unadjusted estimates				Adjusted estimates			
Age-group, years	1999-2000	2003-2004	2009-2010	2017-2018	1999-2000	2003-2004	2009-2010	2017-2018
25–54								
Metro	5.6** (5.4-5.9)	5.9 (5.8-6.0)	5.5 (5.4-5.6)	6.0 (5.9-6.2)	5.6** (5.5-5.8)	6.0 (5.9-6.1)	5.5 (5.5-5.6)	6.0 (5.9-6.2)
Medium-Small	6.5*** (6.3-6.7)	7.2 (7.1-7.4)	7.1 (7.0-7.2)	8.4 (8.2-8.5)	6.4*** (6.2-6.6)	7.0 (6.9-7.2)	6.9 (6.8-7.0)	8.2 (8.1-8.3)
Rural	7.2*** (6.9-7.5)	8.4 (8.1-8.6)	8.8 (8.6-9.0)	11.2 (10.9-11.5)	7.1*** (6.8-7.4)	8.0 (7.8-8.2)	8.4 (8.2-8.5)	10.7 (10.4-11.0)
55–74								
Metro	60.6*** (59.6-61.6)	54.3 (53.8-54.8)	43.4 (42.8-44.0)	45.7 (45.1-46.3)	61.2*** (60.3-62.2)	55.3 (54.9-55.8)	44.1 (43.5-44.6)	46.4 (45.9-47.0)
Medium-Small	62.3*** (61.0-63.5)	58.7 (58.1-59.4)	48.4 (47.7-49.1)	53.5 (52.9-54.2)	61.9*** (60.7-63.1)	57.9 (57.2-58.6)	48.0 (47.5-48.6)	53.1 (52.5-53.7)
Rural	63.3 (61.5-65.1)	61.5 (60.4-62.6)	53.3 (52.4-54.3)	61.9 (60.6-63.2)	62.7[a] (60.8-64.7)	60.1 (59.1-61.1)	51.7 (51.0-52.4)	60.3 (59.2-61.4)
≥75								
Metro	206.4*** (202.8-210.0)	204.1 (201.7-206.5)	179.5 (176.9-182.1)	166.4 (163.1-169.7)	213.3*** (209.4-217.2)	210.2 (207.9-212.5)	183.5 (181.4-185.6)	171.5 (167.9-175.2)
Medium-Small	218.9*** (215.0-222.9)	221.5 (219.2-223.9)	192.6 (190.2-194.9)	177.6 (173.9-181.3)	219.9*** (216.6-223.3)	224.0 (221.7-226.4)	196.5 (194.4-198.7)	182.0 (178.1-185.9)
Rural	238.6*** (232.1-245.1)	245.0 (240.7-249.2)	219.7 (215.8-223.6)	207.9 (202.5-213.3)	234.5*** (229.7-239.4)	244.4 (240.3-248.5)	219.4 (216.3-222.6)	208.3 (203.8-212.8)

Estimated annual diabetes mortality rates (ADMR [95% confidence interval]; per 100,000 people) from unadjusted and adjusted estimates that included sex, region, and up to 3-way interaction terms (age-group, county urbanization, year).
Statistical significance at [a]P<.05; **P<0.001; ***P<0.0001 compared to 2017–2018.

© 2022 Dugani SB et al. *JAMA Network Open.*

eTable 8. Estimated Annual Diabetes Mortality Rates (ADMR), 1999–2018, by Region and County Urbanization, for Diabetes as the Underlying Cause of Death

	**Estimated annual diabetes mortality rate, per 100,000 people**							
	Unadjusted estimates				Adjusted estimates			
Region	1999-2000	2003-2004	2009-2010	2017-2018	1999-2000	2003-2004	2009-2010	2017-2018
Northeast								
Metro	38.1 (37.3-39.0)	35.4 (35.0-35.9)	29.8 (29.4-30.2)	29.2*** (28.5-30.0)	37.2 (36.4-38.0)	34.5 (34.1-35.0)	29.0 (28.6-29.4)	28.5*** (27.8-29.2)
Medium-Small	41.8 (40.8-42.7)	40.2 (39.5-40.9)	34.8 (34.2-35.3)	35.0*** (34.2-35.7)	37.9 (37.0-38.8)	36.5 (35.9-37.1)	31.5 (31.0-32.0)	31.8*** (31.2-32.5)
Rural	47.9 (46.3-49.5)	47.1 (46.1-48.0)	41.8 (41.1-42.6)	43.7** (42.1-45.2)	40.9 (39.5-42.3)	40.2 (39.4-41.0)	35.7 (35.1-36.4)	37.5** (36.1-38.8)
Midwest								
Metro	41.6 (40.6-42.5)	39.0 (38.6-39.5)	32.7 (32.3-33.0)	31.9*** (31.2-32.6)	43.4 (42.4-44.4)	40.8 (40.3-41.3)	34.1 (33.7-34.5)	33.3*** (32.6-34.0)
Medium-Small	44.6 (43.6-45.6)	43.7 (43.1-44.4)	38.0 (37.6-38.4)	38.7*** (38.1-39.3)	42.7 (41.7-43.7)	41.9 (41.3-42.5)	36.4 (36.0-36.8)	37.2*** (36.6-37.8)
Rural	50.4 (49.1-51.8)	50.9 (50.1-51.7)	45.9 (45.3-46.5)	48.6[a] (47.6-49.7)	42.1 (41.0-43.2)	42.4 (41.8-43.1)	38.3 (37.8-38.8)	41.1 (40.2-41.9)
South								
Metro	38.4 (37.7-39.1)	36.3 (35.9-36.7)	29.8 (29.5-30.1)	28.6*** (28.1-29.0)	43.0 (42.2-43.7)	40.6 (40.1-41.0)	33.3 (33.0-33.7)	31.8*** (31.3-32.3)
Medium-Small	46.8 (45.8-47.8)	46.1 (45.6-46.7)	39.5 (39.1-39.8)	39.8*** (39.1-40.5)	44.5 (43.5-45.4)	43.9 (43.4-44.4)	37.6 (37.3-37.9)	38.0*** (37.4-38.6)
Rural	52.6 (50.8-54.4)	53.8 (52.8-54.9)	48.7 (48.1-49.4)	53.1 (52.1-54.1)	47.7 (46.1-49.4)	49.0 (48.0-50.0)	44.5 (43.9-45.1)	48.7 (47.8-49.7)
West								
Metro	30.8 (29.8-31.7)	31.4 (31.0-31.9)	28.6 (28.2-29.0)	30.9 (30.2-31.6)	34.2 (33.1-35.3)	34.9 (34.4-35.4)	31.7 (31.3-32.2)	34.2 (33.5-35.0)
Medium-Small	37.5 (36.6-38.4)	38.4 (37.9-38.9)	34.2 (33.8-34.6)	35.8* (35.0-36.6)	37.9 (37.0-38.8)	38.8 (38.3-39.3)	34.6 (34.2-35.0)	36.1* (35.3-36.9)
Rural	44.1 (42.6-45.5)	44.8 (44.0-45.6)	39.3 (38.7-39.9)	40.4*** (39.4-41.4)	40.3 (39.0-41.6)	41.0 (40.3-41.8)	36.1 (35.5-36.6)	36.9*** (36.0-37.8)

Estimated annual diabetes mortality rates (ADMR [95% confidence interval]; per 100,000 people) from unadjusted and adjusted estimates that included sex, age-group, up to 3-way interaction terms (region, county urbanization, year), and age-standardized to the 2009–2010 population.
Statistical significance at [a]P<.05; *P<0.01; **P<0.001; ***P<0.0001 compared to 2017–2018.

© 2022 Dugani SB et al. *JAMA Network Open.*
